# Impact of Seizure Burden in Hypoxic Ischemic Encephalopathy

**DOI:** 10.15844/pedneurbriefs-29-10-1

**Published:** 2015-10

**Authors:** Andrea C. Pardo

**Affiliations:** 1 Division of Neurology, Ann & Robert H. Lurie Children’s Hospital of Chicago, Chicago, IL; 2 Departments of Pediatrics and Neurology, Northwestern University Feinberg School of Medicine, Chicago, IL

**Keywords:** Seizure Burden, Hypoxic Ischemic Encephalopathy, Neurodevelopmental Outcome

## Abstract

Investigators from Washington University St. Louis studied the impact of electroencephalographic monitoring of neonates with Hypoxic Ischemic Encephalopathy (HIE) and their degree of MRI injury and neurodevelopmental outcomes when increasing seizure burden was present.

Investigators from Washington University St. Louis studied the impact of electroencephalographic monitoring of neonates with Hypoxic Ischemic Encephalopathy (HIE) and their degree of MRI injury and neurodevelopmental outcomes when increasing seizure burden was present. Investigators performed a randomized controlled trial to investigate the effect of seizure burden on neurodevelopmental outcomes in infants with recognized electrographic seizures versus infants with clinically recognized seizures. Researchers found 15/35 neonates in the electrographic seizure group (ESG) and 20/34 neonates in the clinical seizure group (CSG) to have seizures. The researchers found that the ESG presented with a lower cumulative seizure burden (SB) than the CSG (p=0.02) likely due to faster identification and subsequent treatment of seizures. The investigators found no difference between ESG and CSG groups regarding developmental outcome due to the power of the study. However, analysis of the cohort as a whole indicated that an increasing SB correlated with worsened outcomes on the Bayley Scales of Infant Development III (p=0.03). The investigators also showed that greater SB in the cohort was associated with a worse MRI injury severity. [1]

COMMENTARY. Neonatal seizures are prevalent in infants with HIE [2]. However, they are difficult to diagnose clinically since most seizures in neonates are subclinical [3]. This study highlights the need for electrographic monitoring of infants in accordance to the American Clinical Neurophysiology Society guidelines [4]. Although this study does not show a difference among groups monitored clinically or electroencephalographically due to the power of the study, it shows that greater cumulative seizure burden may correlate with poor neurodevelopmental outcomes and worse MRI injury scoring. Further prospective studies are necessary to establish whether treatment of seizures and interventions to reduce seizure burden have an impact on neurodevelopmental outcome in infants with HIE.
